# Smart Pellets for Controlled Delivery of 5-Fluorouracil

**DOI:** 10.3390/molecules28010306

**Published:** 2022-12-30

**Authors:** Mohammad F. Bayan, Abdolelah Jaradat, Mohammad H. Alyami, Abdallah Y. Naser

**Affiliations:** 1 Faculty of Pharmacy, Philadelphia University, P.O. Box 1, Amman 19392, Jordan; 2 Faculty of Pharmacy, Isra University, P.O. Box 33, Amman 11622, Jordan; 3 Department of Pharmaceutics, College of Pharmacy, Najran University, Najran 66462, Saudi Arabia

**Keywords:** 5-fluorouracil, sustainable materials, smart polymers, specific drug delivery, colorectal carcinoma

## Abstract

This work aimed to develop a new one-pot and readily scaled-up formulation capable of retaining 5-fluorouracil and prolonging its release to obtain a site-specific medication delivery for the potential treatment of colorectal cancer. Six polymer-based formulations were successfully produced using a thermal bulk polymerization method and loaded with 5-fluorouracil, which is a chemotherapeutic agent used in the treatment of colorectal carcinoma. The pellets produced were characterized by measuring the glass transition temperature, tensile strength, Young’s modulus, and tensile elongation at break. Studies on in vitro swelling and release were carried out in phosphate-buffered saline to evaluate the behaviour of the developed system. The Young’s modulus, glass transition temperature, and tensile strength all increased significantly as the crosslinker concentration increased, but the fracture strain value reduced significantly. The in vitro swelling profile of the produced formulations was significantly reduced by increasing crosslinking density. Less than 27% cumulative drug release was achieved for all formulations after 5 h of starting the release study. The highest cumulative drug release reached after 24 h was 69%. The developed drug delivery system demonstrated the ability to delay the release of 5-fluorouracil in upper gastrointestinal tract-mimicking conditions, while permitting its release in a controlled way afterward, which makes it promising for the potential delivery of 5-fluorouracil to the colon.

## 1. Introduction

Targeted drug delivery to the colon has attracted the attention of many researchers owing to its tremendous advantages over systemic drug administration, including increased drug potency and efficacy and reduced adverse drug reactions [1,2]. Colonic drug delivery systems have been used to treat a plethora of bowel diseases encompassing colorectal carcinoma, ulcerative colitis, diverticulitis, Crohn’s disease, and irritable bowel syndrome [3,4,5]. Colon-specific drug delivery has long been used to enhance therapeutic effectiveness by reducing unwanted absorption in other regions of the gastrointestinal tract, guaranteeing that the whole drug dose is specifically delivered to the site of interest in the colon [6,7]. Most colon-targeted drug delivery systems are either responsive to the pH of the colon or to enzymes produced by intestinal microbiota [8,9,10]. For instance, metronidazole, which is used for treatment of bowel infection, was formulated in a tablet containing pH-sensitive polymers consisting of Eudragit E, Eudragit L, and alginate in order to delay the release of metronidazole and achieve a once daily formulation [11]. In this study, metronidazole release was sustained for 12 h; however, a percentage of the drug was released in acidic medium (simulated gastric fluid). Moreover, a combined time-dependent and pH-sensitive multilayer system was composed of an outer layer consisting of a pH-sensitive polymer that degrades at pH greater than 5, a middle layer containing swellable HPMC polymer, and an inner layer composed of enteric coating material [12]. The system has shown colon targeting capability, as demonstrated by in vivo rat experiments. Additionally, chitosan nanoparticles have been loaded with the immunosuppressive drug tacrolimus for inflammatory bowel disease targeting [13]. The nanoparticles were coated with a pH-sensitive Eudragit S 100 polymer and hyaluronic acid as a targeting ligand for CD44 receptors expressed by leukocytes at the site of inflamed intestine, and the results showed significant reduction in the inflammatory mediators produced by macrophages. Colon-specific drug delivery has also been utilized for the treatment of colorectal carcinoma diseases by specifically delivering chemotherapeutic agents to the colon using polymeric carriers [14]. For example, alginate microparticles were coated with Eudragit S 100 for colon-targeted delivery of 5-fluorouracil (FU), which is a chemotherapeutic agent used for treatment of colorectal cancer [15]. The release studies have shown almost zero drug release in the first 4 h in simulated gastric fluid; additionally, the release of the drug was sustained for 20 h, indicating the suitability of this system for colon-specific delivery of anti-cancer agents. Several drug chemical modification techniques have been employed to specifically deliver drugs to the colon, such as using prodrugs, which are pharmacologically inactive substances; however, they are activated when they reach the colon [16,17]. For instance, azo-based compounds composed of azo groups covalently attached to anti-inflammatory drugs used for irritable bowel diseases have been utilized for colon targeting of these drugs, where the resultant prodrugs become specifically active in the colon due to the reducing effect of azoreductase produced by gut microbiota cleaving the bond and release the drug to the site of interest [18]. Nagpal et al. synthesized an azo-based prodrug of 5-amino salicylic acid by chemically conjugating l-histidine to 5-amino salicylic acid via azo linkage, where the drug release was minimal in simulated gastric fluid and only 14 percent of the drug released in simulated intestinal fluid without the bacterial azoreductase. However, more than 85 drugs were released in a medium consisting of rat faeces, which contains intestinal microbiota, indicating azoreductase-responsive drug release in the colon [19]. Another example is conjugating l-alanine to 5-salicylic acid for colon targeting, and the results demonstrated that the prodrug was specifically in the gut microbiota of the rabbit via oral and intercaecal routes, indicating successful colon-specific delivery [20]. Moreover, colonic drug delivery is not only used for localized treatment of gut diseases, but also for oral delivery of protein therapeutics, which are acid labile and enzyme degradable, to protect these therapeutics from the harsh gastrointestinal conditions preceding the absorption process [21]. For example, uricase, an enzyme responsible for breaking down urate crystals accumulated in body joints, was encapsulated into alginate/pectin microparticles for the treatment of gout [22]. These microparticles were coated with a pH-responsive polymer (Eudragit S-100) to protect the protein (uricase) from degradation by gastric enzymes, where this system displayed enhanced colonic absorption when the drug was combined with bile salts. It has also been reported that aloe vera (*A. vera*) gel was employed for insulin delivery to the colon to prevent its gastrointestinal degradation [23]. In this study, ex vivo experiments demonstrated that insulin absorption was maximally promoted at the colon region compared to the ilium and jejunum, indicating effective colon targeting using *A. vera*. Moreover, recent advances have focused on oral delivery of human insulin using mucus penetrating PLGA-nanoparticles, which successfully protect insulin from the acidic environment of simulated gastric fluid and enhance its absorption across intestinal epithelial cells [24]. Most previous studies have demonstrated the importance of colon-specific drug delivery systems; however, they involved multi-step processes for the synthesis of drug delivery carriers. Therefore, this research aims to design a new one-pot formulation composed of methacrylate derivatives for the delivery of FU in order to treat colorectal carcinoma. This polymer has the capability to retain a drug and prolong its release to enable site-specific drug delivery to the colon only. The synthetic procedure can also be readily scaled up for continuous manufacturing.

## 2. Results and Discussion

### 2.1. Pellet Production Using Polymeric Materials

FU is a chemotherapeutic medication used, alone or in combination with other anti-cancer agents, in the treatment of many cancers affecting the cervix, head, neck, and gastrointestinal tract. The mechanism of action of FU is believed to be via the inhibition of thymidylate synthase [15,16]. The main goal of this work was to develop a new one-pot formulation capable of retaining FU and prolonging its release to elicit a site-specific FU delivery for the potential treatment of colorectal carcinoma. The formulation developed was based on hydroxyethyl methacrylate (HA) copolymerized with methacrylic acid (MA). Six formulations (Table 6) were produced successfully using a thermal bulk polymerization method and loaded with FU. This system was optimized and designed to selectively deliver FU to the colon by utilizing an effective time-/pH-dependent approach. It is based on preventing/delaying the FU release within the first 5–6 h following its oral administration to ensure FU arrival to the colonic region. This can enhance the therapeutic outcome of the regimen, reduce dosing/unwanted side effects, and increase patient compliance. A satisfactory drug entrapment efficiency (DE%) of about 91% was obtained in the drug-loaded formulations (Table 1).

### 2.2. Thermal Characterization

The dynamic mechanical thermal (DMT) instrument is commonly used in the thermal characterization of polymers’ viscoelasticity and to determine the glass transition temperature (Tg) value. At Tg, the polymer undergoes a reversible transformation from a rigid to a flexible state. It is an important indicator of the polymer chains’ flexibility. Tg increases as the polymer’s rigidity increases. Thermal characterization was performed to determine the Tg of the pellets developed (Table 2). The Tg values were in the 121–128 °C range. The employment of FU has a negligible impact on the polymer’s Tg value, as demonstrated by the Tg values of M4–M6. As the crosslinker (EA) concentration increased, the Tg value of the polymer increased significantly. This can be attributed to the decreased elasticity of the polymer and the development of a more rigid structure.

### 2.3. Mechanical Characterization

The mechanical properties of polymers are important in describing the physical behaviour of the materials under different external stresses, which reflects the elasticity and rigidity of the polymer. The stress–strain curve represents a simple method to determine the mechanical properties of polymers. The ultimate tensile strength (TS) refers to the force necessary to fracture the polymer, Young’s modulus (YM) refers to the material’s overall rigidity, and fracture strain (TE) is the maximum strain the material can withstand before breaking. The pellets’ mechanical properties (TS, YM, and TE), obtained from the stress–strain curve, are shown in Table 3. The stress-strain curves of all formulations are presented in the Supplementary Materials (Figures S1–S6). FU employment had a negligible impact on the polymer’s mechanical characteristics. As the crosslinker-ethylene glycol dimethacrylate (EA) concentration increased, the TS and YM values significantly increased, while the TE significantly decreased. This is derived from the fact that raising the crosslinking density causes the formation of a more rigid polymeric structure.

### 2.4. Pellets’ Swelling Assessment

**Figure 1:**
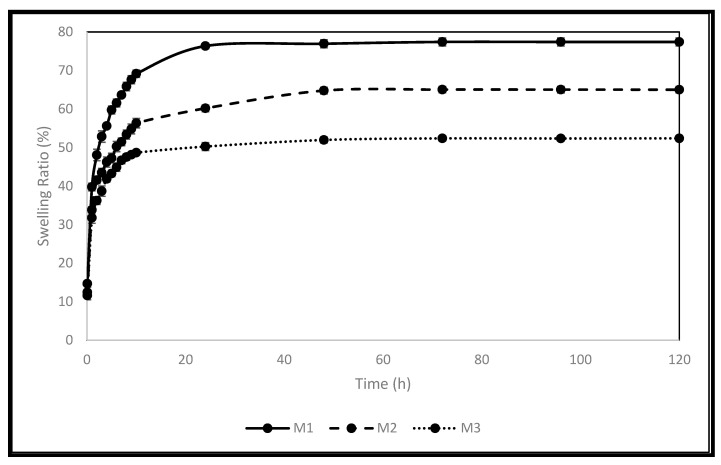
The produced pellets’ swelling behaviour in PBS.

**Figure 2:**
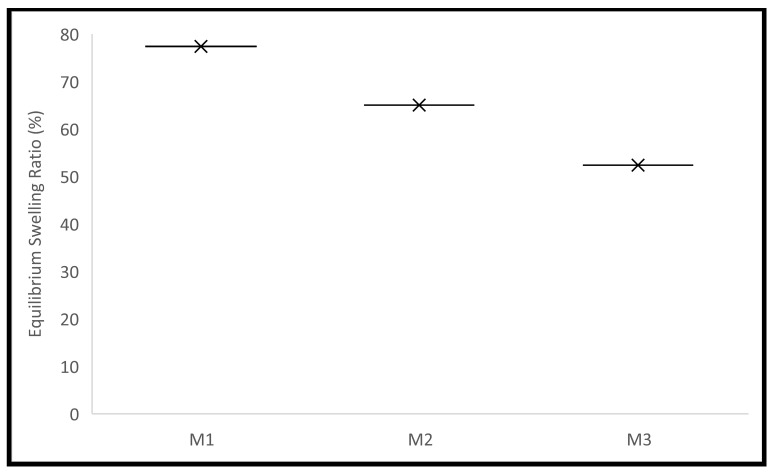
The produced pellets’ equilibrium swelling in PBS.

Polymer’s swelling is an intrinsic feature, where it enlarges due to the penetration of fluid into the void space between the polymeric chains. This behaviour can be influenced by some factors related to the surrounding environment of the polymer, such as temperature, pH, and ionic strength. The crosslinking density can affect this behaviour, as it influences the available voids. The size of the polymer also affects this behaviour, as it affects the surface area available for fluid penetration. The ability to control this behaviour encourages the use of polymers in many pharmaceutical drug delivery systems [1,2,3,4,5,6,7]. The pellets’ swelling was examined in phosphate-buffered saline (PBS). The equilibrium selling ratio and in vitro swelling behaviour of the produced formulations are shown in Figure 1 and Figure 2. The swelling rate constants (k) of each formulation are shown in Table 4 following the application of the Korsmeyer-Peppas model (Supplementary Materials: Figure S7). Increasing the crosslinker (EA) concentration decreased the pellets’ swelling profile and rate significantly, with equilibrium ratios of 77%, 65%, and 52% achieved for M1, M2, and M3, respectively. This can be explained by the decreased elasticity of the polymer and the increase in rigidity because of the increased EA concentration [7]. Enhanced electrostatic repulsions between the pendant groups are due to the presence of the ionizable anionic pendant group (MA) in these pellets. This ionization is triggered in the simulated intestinal fluid (PBS), where the pH is higher than the pKa of MA, thus contributing to the high swelling ratios of the pellets produced [4]. The equilibrium swelling ratios were attained within 72 h for all pellets. The results obtained are in agreement with the data published recently by Bayan et al. [4] concerning the polymer used.

### 2.5. In Vitro Release Study

**Figure 3:**
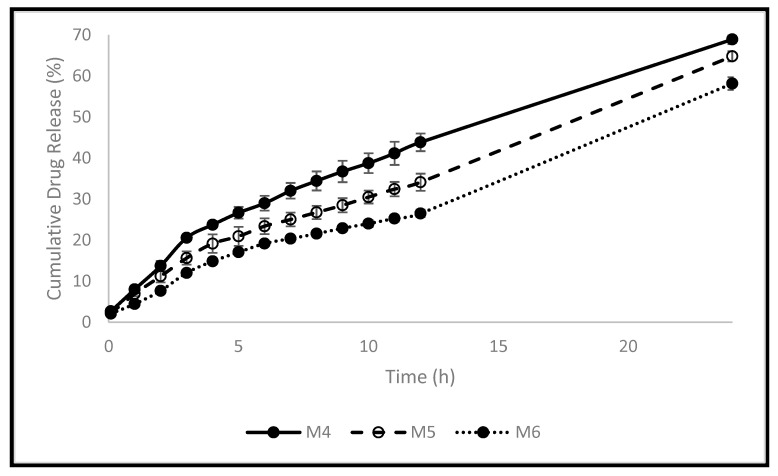
The pellets’ release profiles in PBS.

Controlled drug delivery systems based on polymers have been fabricated to facilitate and deliver the medication to their sites of action within the body. This can enhance the medication’s efficacy and safety, as well as patient compliance. The release profile of FU from the polymeric pellets developed was investigated in PBS (Figure 3). Table 5 displays the swelling rate constants (k), R2, and n values following the application of the Korsmeyer-Peppas model (Supplementary Materials: Figure S8). As the crosslinker (EA) concentration increased, the release profile of FU decreased significantly, which is consistent with thermal, mechanical, and in vitro swelling studies because of the reduction of the polymer’s elasticity and the development of a more rigid structure [7]. After 5 h, less than 27% cumulative release was achieved for all pellets (26.5%, 21.0%, and 17.0% for M4, M5, and M6, respectively). After 12 h, 44.0%, 34.0%, and 26.5% cumulative drug release was obtained for M4, M5, and M6, respectively. M4 achieved the highest cumulative drug release after 24 h (69%) compared to 65% and 58% for M5 and M6, respectively. This can be explained by the decreased elasticity of the polymer and the increase in rigidity because of increasing EA concentration in M4 compared to that in M5 and M6. An oral drug needs approximately five hours to reach the large intestine. A colon-specific delivery should be designed to postpone or forbid FU release during this period to ensure the drug arrival to the colonic region. The in vitro release profile of FU from the developed pellets demonstrated the potential to delay FU release for the first five to six hours while allowing it to be released in a controlled manner later. This makes it attractive to deliver FU to the colon. To investigate the release rate constant (k) and the mechanism of drug release, the first 60% of the release data were fitted to the Korsmeyer-Peppas model (Equation (1), where the drug release fraction is F, t is time in hours, and n is the release exponent). When a drug is released from a polymeric dosage form, such as a hydrogel, or when the release follows multiple kinetics mechanisms, such as a combination of more than one type of release mechanism, the Korsmeyer-Peppas model is used to describe and analyze the drug release [4]. All pellets had n values of more than 0.5 and less than 1, as seen in Table 5, indicating an anomalous mechanism of drug release. This indicates that diffusion and polymer swelling control the release of FU. The results obtained are in agreement with the data published recently by Bayan et al. [4] concerning the polymer used.

(1) $$F=k\;t^{n}$$

## 3. Materials and Methods

### 3.1. Materials

Methacrylic acid (MA), hydroxyethyl methacrylate (HA), 5-fluorouracil (FU), ethylene glycol dimethacrylate (EA), and azobisisobutyronitrile (AN) were purchased from Sigma-Aldrich (St. Louis, MO, USA). All investigations used ultra-pure water. The materials were used exactly as they were provided and without any alterations.

### 3.2. Methods

#### 3.2.1. Pellet Production Using Polymeric Materials

Six polymeric-based pellets were produced using a thermal bulk polymerization technique (Table 6), based on HA and MA monomers, and loaded with FU as a model medication. AN was used as a thermal initiator. Each formulation resulted in the production of a 10 g polymeric film, which was then pierced into uniform small pellets using a cork borer no. 1 (5 mm). In an amber glass container, the components of each formula were mixed and stirred for 60 min at room temperature. The final mixture was injected, using a 20 mL syringe, into a pre-made. The mould was then placed in an oven that had been set to 60 °C, where the polymerization process took place for 18 h. Two borosilicate glass sheets (215 mm × 215 mm × 3 mm), eight 32 mm foldback clips, and a silicon-coated release liner were used to create the mould. The silicone-coated sheet was placed on top of the glass sheets, and one of the glass sheets was used to sketch the mould’s borders using a silicone tube (0.76 mm × 1.65 mm × 0.445 mm). The other glass sheet was then placed on top of it, and foldback clips were used to hold the two sheets vertically together. Each film was put in a storage container coated with aluminium foil and immersed in ultra-pure water at the end of the synthesis process. Daily water changes were made to wash the produced disc and get rid of unreacted species. The washing procedure was monitored using a UV/VIS spectrophotometer (Spectroscan 80 D, Nicosia, Cyprus). The produced pellets’ drug entrapment efficiency (DE%) was estimated using the formula in Equation (2). The pellets were crushed using a ball milling technique and then placed in PBS (pH 7.4) inside the shaking bath (SWB-A), running at 100 cycles per minute at 37 °C for 24 h. The samples were then filtered, and their absorbance was measured at 266 nm using a UV/VIS spectrophotometer (Spectroscan 80 D). The loaded content serves as a representation of the drug concertation initially loaded in the formulation (5% *w*/*w*).

(2) DE%=Analyzed drug content/Loaded content×100%

#### 3.2.2. Thermal Characterization

A Q800 dynamic mechanical thermal (DMT) analyzer was used to determine the glass transition temperature of the formulations produced. In this method, the probe applies sinusoidal stress on the sample, and deformation caused by this stress is detected and measured. The samples were analyzed at 35–160 °C, 1 Hz, and 3 °C/min. These conditions were selected based on a recently published study by Bayan et al. [4] concerning the polymer used. The tan δ curve’s peak was identified as the glass transition temperature (Tg). Triplicates were carried out. Calculations were made for the mean and standard deviation. One-way analysis of variance was used to statistically examine the data, and Tukey’s multiple comparison test was performed to statistically assess the data (*n* = 3, *p* 0.05).

#### 3.2.3. Mechanical Characterization

A TA-XT plus texture analyzer was used to characterize the mechanical properties of the formulations produced. The dried discs (25 × 10 mm) were secured among the clutches to leave a constant 20 mm length under strain. The upper fastener was operated at a fixed rate (500 µm/s) till breaking the disc. The ultimate tensile strength (TS), Young’s modulus (YM), and fracture strain (TE) were obtained from the stress–strain curve. Triplicates were carried out. Calculations were made for the mean and standard deviation. One-way analysis of variance and Tukey’s multiple comparison test were used to statistically assess the data (*n* = 3, *p* 0.05).

#### 3.2.4. Pellets’ Swelling Assessment

The produced pellets’ in vitro swelling behaviour was assessed in phosphate-buffered saline (PBS, pH 7.4) at 37 °C in a dynamic water bath (Biobase thermostatic shaking bath-SWB-A, Jinan, China). Triplicates of each formulation were weighed and put into amber glass vials. The PBS buffer was maintained in the bath at 37 °C. Each vial received 10 mL of PBS buffer. The pellets were removed from the vials using forceps at predefined intervals, laid out on a thick piece of medical tissue, carefully blotted before being weighed, and put back into the vials in the thermostatic shaking bath. Equation (3) was used to determine the ratio at each time point. These ratios were then plotted against the time to investigate the swelling behaviour of the produced pellets. The Korsemeyer-Peppas model was fitted to the first 60% to determine the rate of swelling for each formulation. This model was created as a simple relationship to identify the swelling rate process in polymeric-based systems. A two-way analysis of variance test was used to statistically assess all data, and Tukey’s multiple comparison test was used to statistically assess the data (*n* = 3, *p* 0.05).

(3) $$Swelling\;ratio\;\left(\%\right)=\left[\frac{The\;weight\;of\;the\;swollen\;pellet-The\;inital\;weight\;of\;the\;dried\;pellet}{The\;weight\;of\;the\;swollen\;pellet}\right]\times 100\%$$

#### 3.2.5. In Vitro Release Study

**Figure 4:**
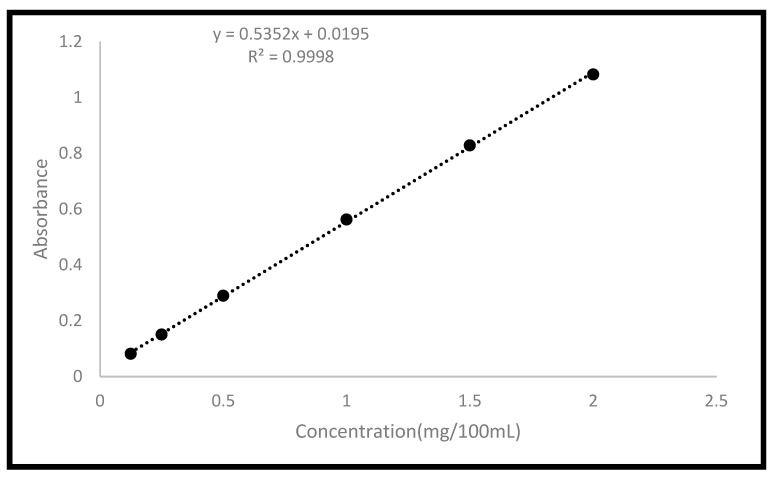
Calibration curve of FU in PBS.

Using a modified Heelan and Corrigan technique, the in vitro release of the model drug (FU) was examined in PBS (pH 7.4) inside the shaking bath (SWB-A), running at 100 cycles per minute at 37 °C [25]. Triplicates of each formulation were carried out and put into 28 mL McCartney bottles. The PBS buffer was previously maintained in the bath at 37 °C. In total, 20 mL of PBS was added to each bottle. At predefined time intervals, 0.5 mL of the sample was removed and replaced with 0.5 mL of fresh PBS. The withdrawn samples were filtered, and their absorbance was measured at 266 nm using a UV/VIS spectrophotometer (Spectroscan 80 D). A fully validated calibration curve was constructed for FU in PBS (Figure 4). The Korsemeyer-Peppas model was fitted to the first 60% data to investigate the rate and mechanism of drug release. This model was developed as a straightforward relationship to detect the mechanism of drug release from polymeric-based systems. A two-way analysis of variance test was used to statistically assess all data, and Tukey’s multiple comparison test was used to statistically assess the data (*n* = 3, *p* 0.05).

## 4. Conclusions

This study investigated the development of a new one-pot formulation composed of methacrylate derivatives for the delivery of FU in order to treat colorectal carcinoma. This can enhance the therapeutic outcome of the regimen, reduce dosing/unwanted side effects, and increase patient compliance. Six polymer-based pellets were produced successfully using a thermal bulk polymerization technique based on the HA and MA monomers, and loaded with FU as a model medication. A satisfactory drug entrapment efficiency of about 91% was obtained in the drug-loaded formulations. The Young’s modulus, glass transition temperature, and tensile strength all increased significantly with the EA concentration, but the tensile elongation at the break value decreased significantly. The in vitro swelling behaviour of the pellets reduced significantly by increasing the crosslinker concentration. Less than 27% cumulative drug release was achieved for all formulations after 5 h of starting the release study. The highest cumulative drug release reached after 24 h was 69%. The developed drug delivery system demonstrated the ability to delay the release of 5-fluorouracil in upper gastrointestinal tract-mimicking conditions, while permitting its release in a controlled way afterward, which makes it promising for the potential delivery of 5-fluorouracil to the colon.

## Figures and Tables

**Table 1:** DE% Drug entrapment efficiency of the drug-loaded pellets.

Formula	DE%
M4	91.44 ± 0.62
M5	91.29 ± 1.63
M6	90.87 ± 1.27

**Table 2:** Thermal characterization of the pellets produced.

Formula	Tg (°C)
M1	121.6 ± 0.94
M2	123.4 ± 1.14
M3	126.8 ± 0.69
M4	121.9 ± 1.05
M5	124.3 ± 1.02
M6	127.3 ± 0.87

**Table 3:** Mechanical characterization of the pellets produced.

Formula	TS (MPa)	YM (MPa)	TE (%)
M1	4.79 ± 0.22	25.28 ± 1.04	3.48 ± 0.21
M2	5.15 ± 0.16	27.12 ± 0.70	2.77 ± 0.14
M3	5.29 ± 0.36	32.61 ± 0.88	2.22 ± 0.29
M4	4.97 ± 0.21	26.29 ± 0.65	3.37 ± 0.17
M5	5.12 ± 0.36	28.74 ± 0.72	2.43 ± 0.16
M6	5.52 ± 0.20	33.01 ± 1.16	1.83 ± 0.33

**Table 4:** The Korsmeyer-Peppas model’s rate constants (k) and R^2^ of the swelling data.

Formula	R^2^	k
M1	0.9868	0.3613
M2	0.9677	0.2962
M3	0.9621	0.2700

**Table 5:** The Korsmeyer-Peppas model’s rate constants (k), n-value, and R^2^ of the release data.

Formulation	R^2^	n	k
M4	0.9885	0.5824	0.1012
M5	0.9931	0.5695	0.0818
M6	0.9611	0.5544	0.0654

**Table 6:** The ingredients of the developed pellets.

Recipe	MA (% wt)	HA (% wt)	EA (% wt)	AN (% wt)	FU (% wt)
M1	20	78	1	1	-
M2	20	74	5	1	-
M3	20	69	10	1	-
M4	20	73	1	1	5
M5	20	69	5	1	5
M6	20	64	10	1	5

## Data Availability

All data are available in the manuscript.

## Supplementary Materials

The following supporting information can be downloaded at: https://www.mdpi.com/article/10.3390/molecules28010306/s1, Figure S1: Strain-stress curves of M1; Figure S2: Strain-stress curves of M2; Figure S3: Strain-stress curves of M3; Figure S4: Strain-stress curves of M4; Figure S5: Strain-stress curves of M5; Figure S6: Strain-stress curves of M6; Figure S7: The swelling data after fitting to the Korsmeyer-Peppas model; Figure S8: The release data after fitting to the Korsmeyer-Peppas model.
