# Supplementary material for: Smart Pellets for Controlled Delivery of 5-Fluorouracil
Source: Molecules. 2022 Dec 30;28(1):306. doi: 10.3390/molecules28010306 (PMC9822034; doi:10.3390/molecules28010306)
Supplement: Supplementary file 1 [file molecules-28-00306-s001.zip › molecules-2089291-supplementary.pdf]

molecules

Supplementary Materials

Smart Pellets for Controlled Delivery of 5-Fluorouracil

Mohammad F. Bayan [1,*], Abdolelah Jaradat [2], Mohammad H. Alyami [3,*] and Abdallah Y. Naser [2]

[1] Faculty of Pharmacy, Philadelphia University, P.O. Box 1, Amman 19392, Jordan
[2] Faculty of Pharmacy, Isra University, P.O. Box 33, Amman 11622, Jordan
[3] Department of Pharmaceutics, College of Pharmacy, Najran University, Najran 66462, Saudi Arabia
* Correspondence: mbayan@philadelphia.edu.jo (M.F.B.); mhalmansour@nu.edu.sa (M.H.A.)

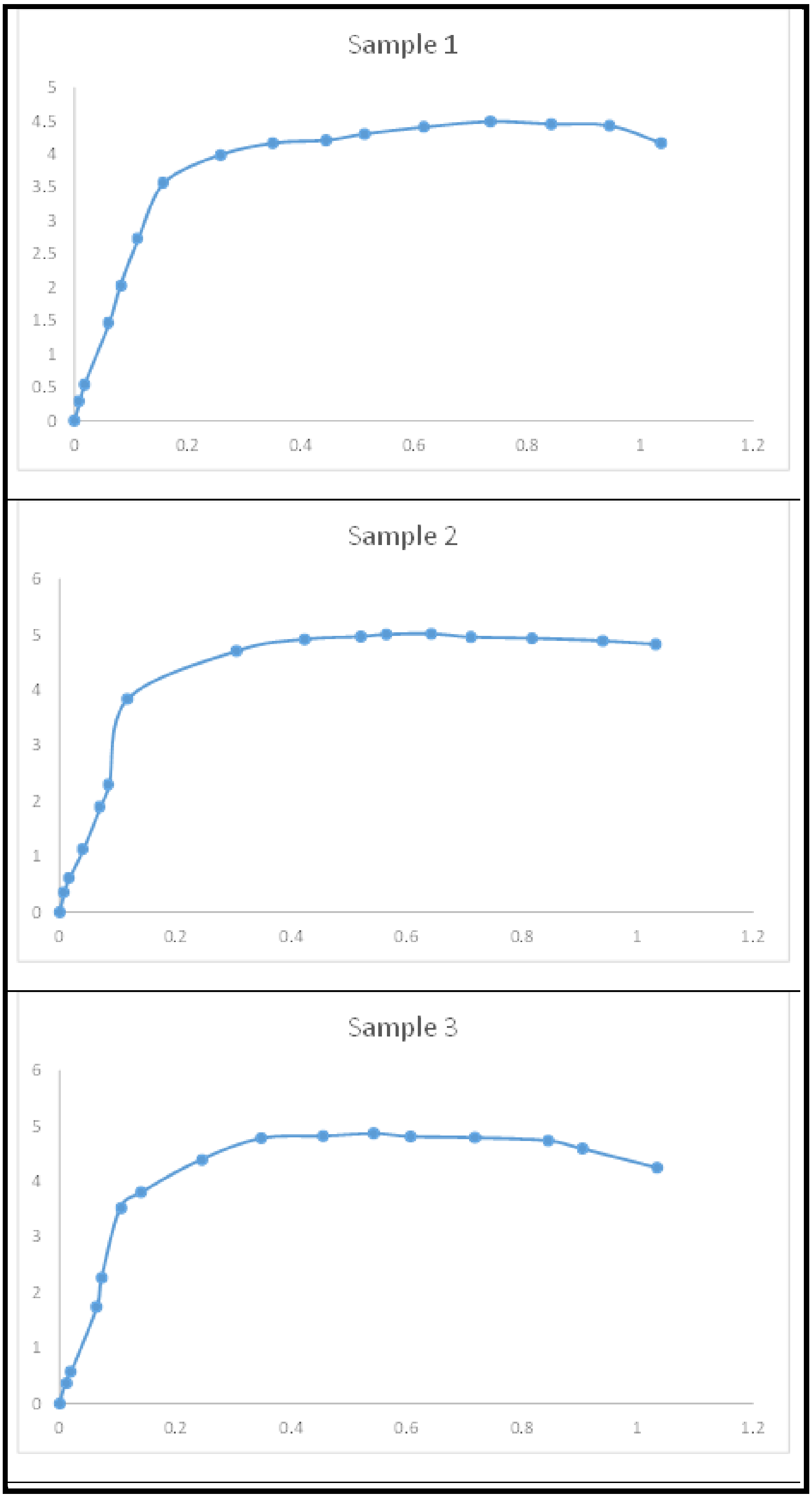

Figure S1. Strain-stress curves of M1

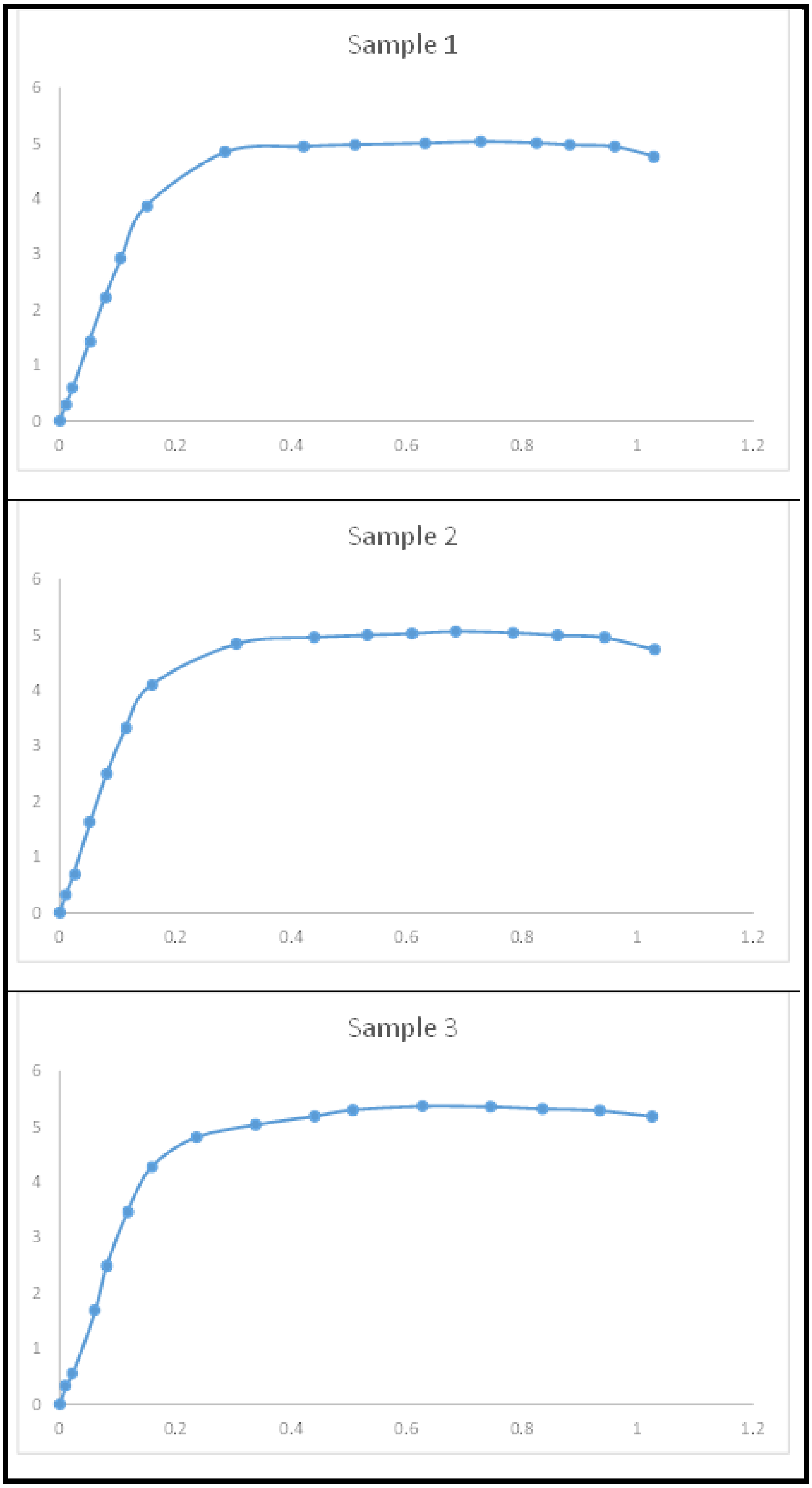

Figure S2. Strain-stress curves of M2

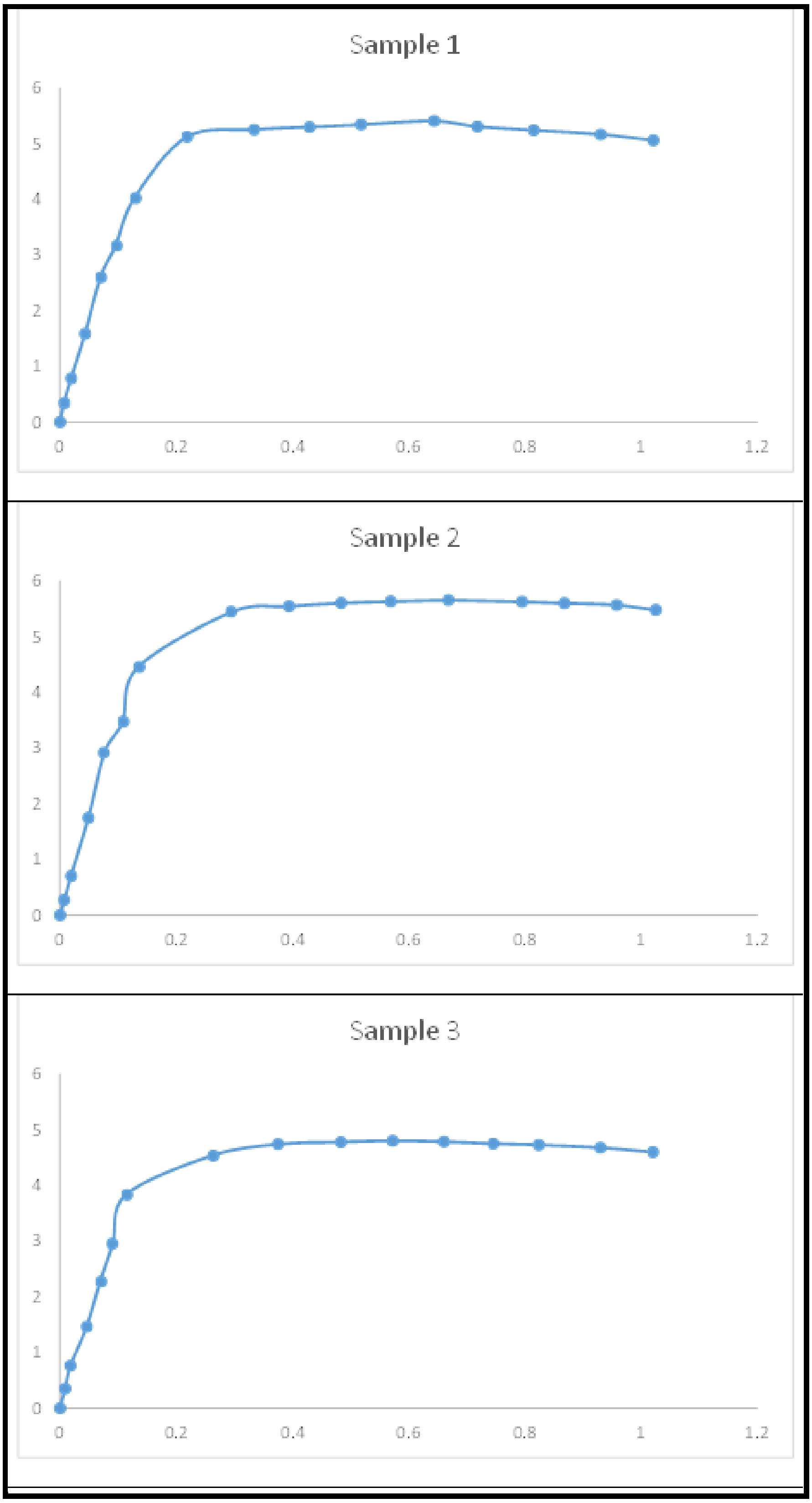

Figure S3. Strain-stress curves of M3

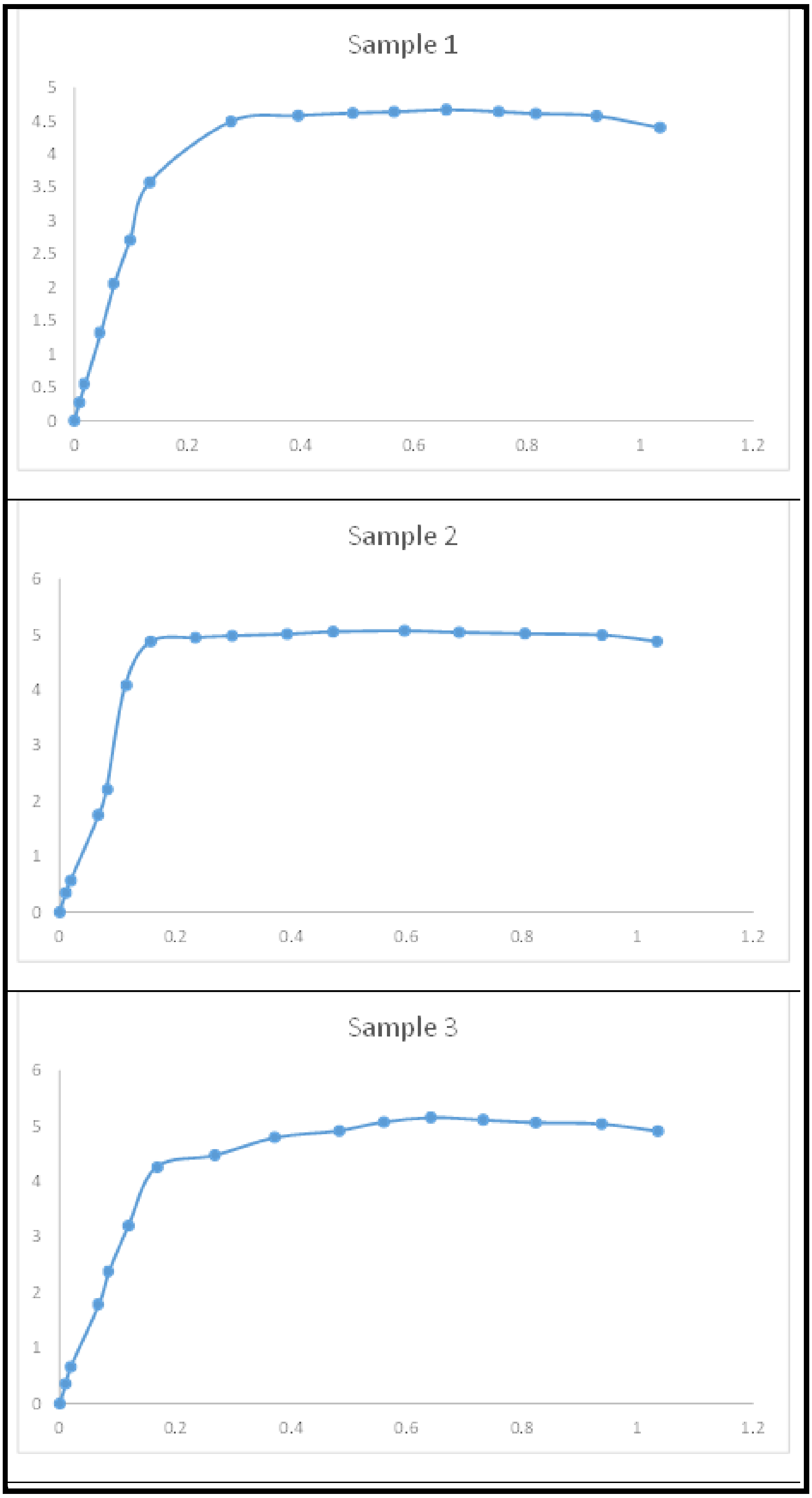

Figure S4. Strain-stress curves of M4

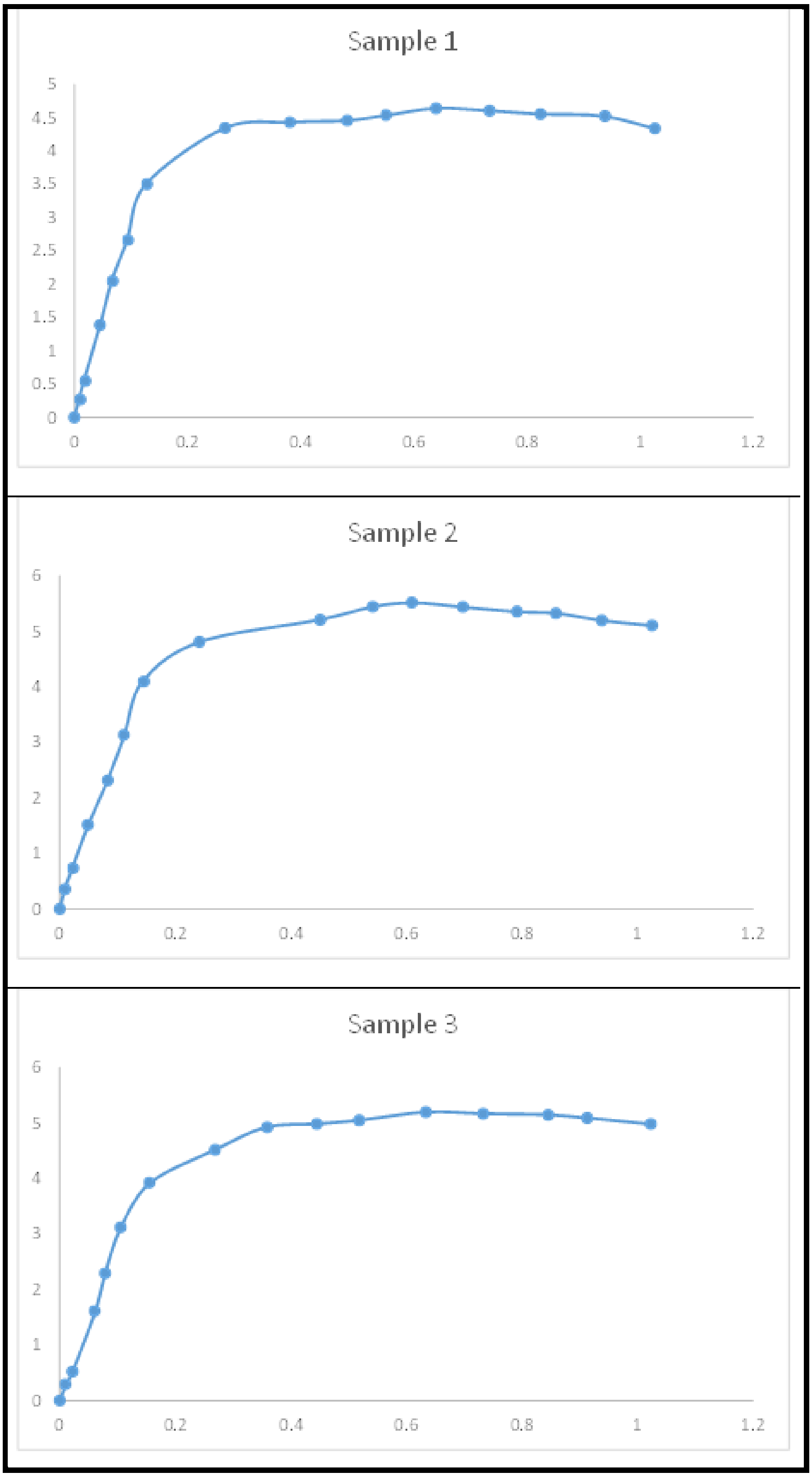

Figure S5. Strain-stress curves of M5

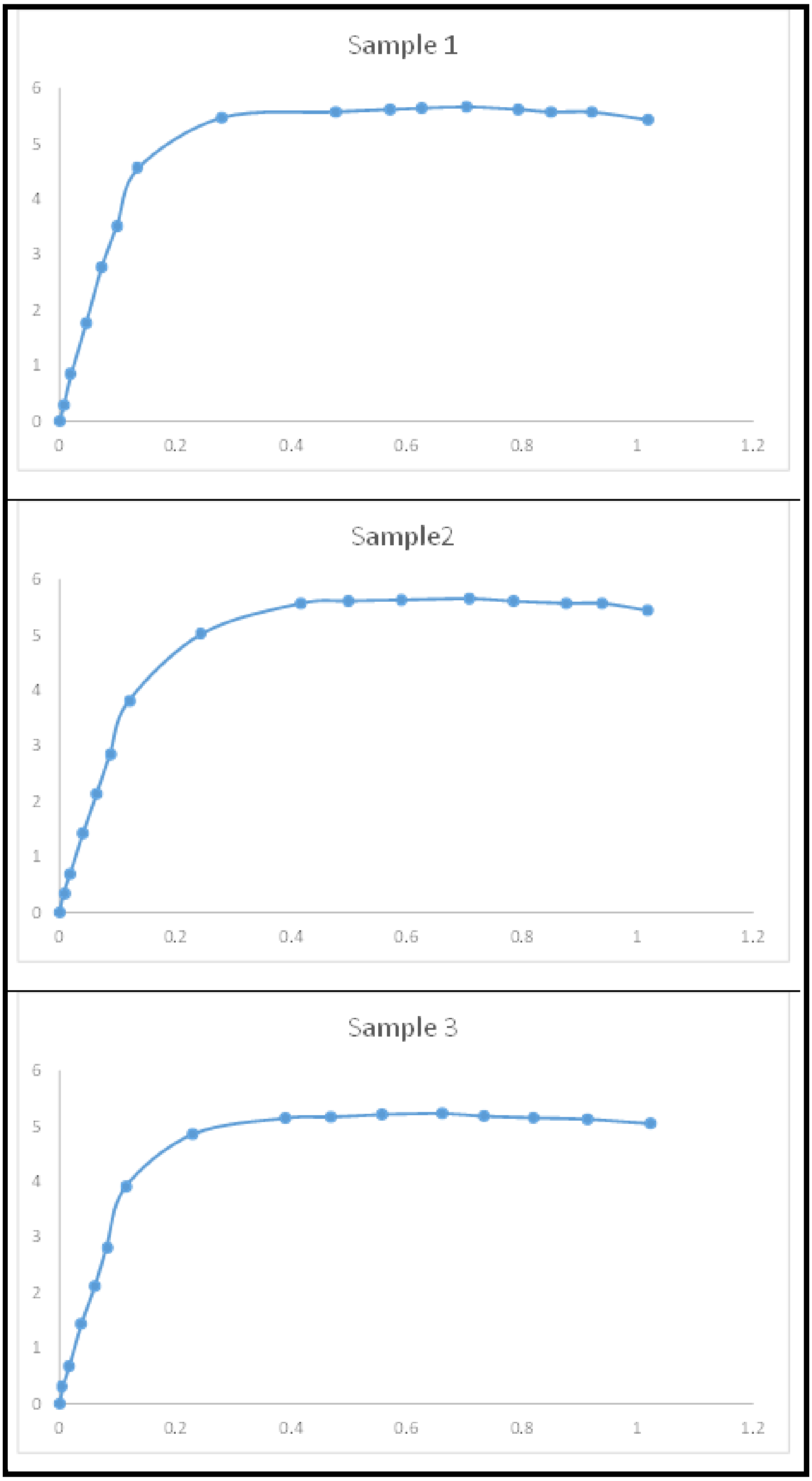

Figure S6. Strain-stress curves of M6

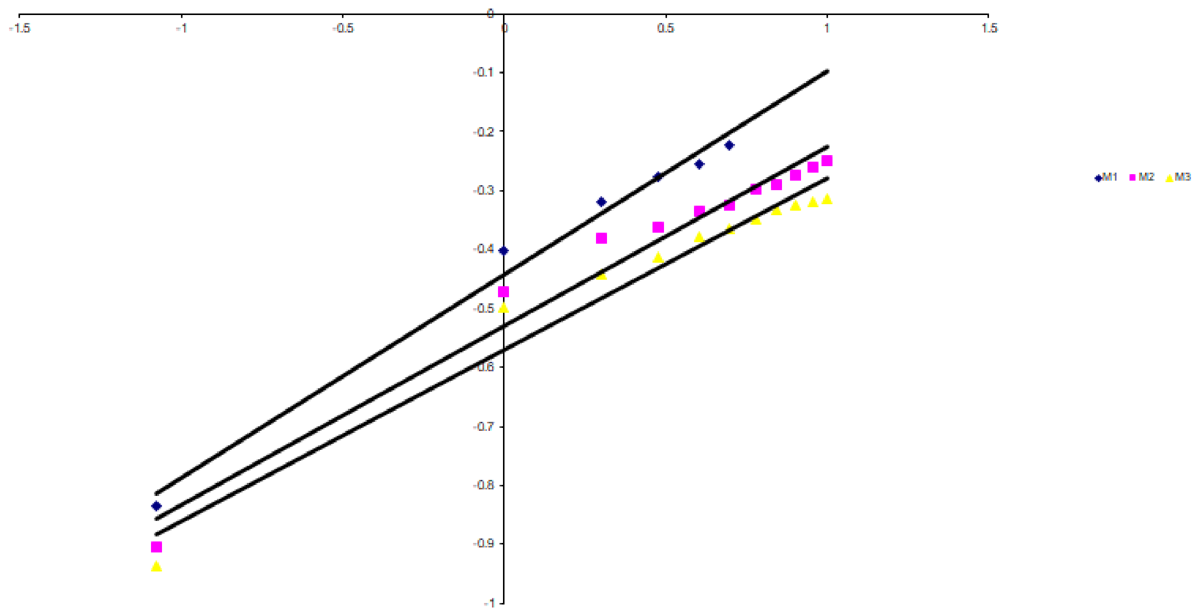

Figure S7. The swelling data after fitting to the Korsmeyer-Peppas model

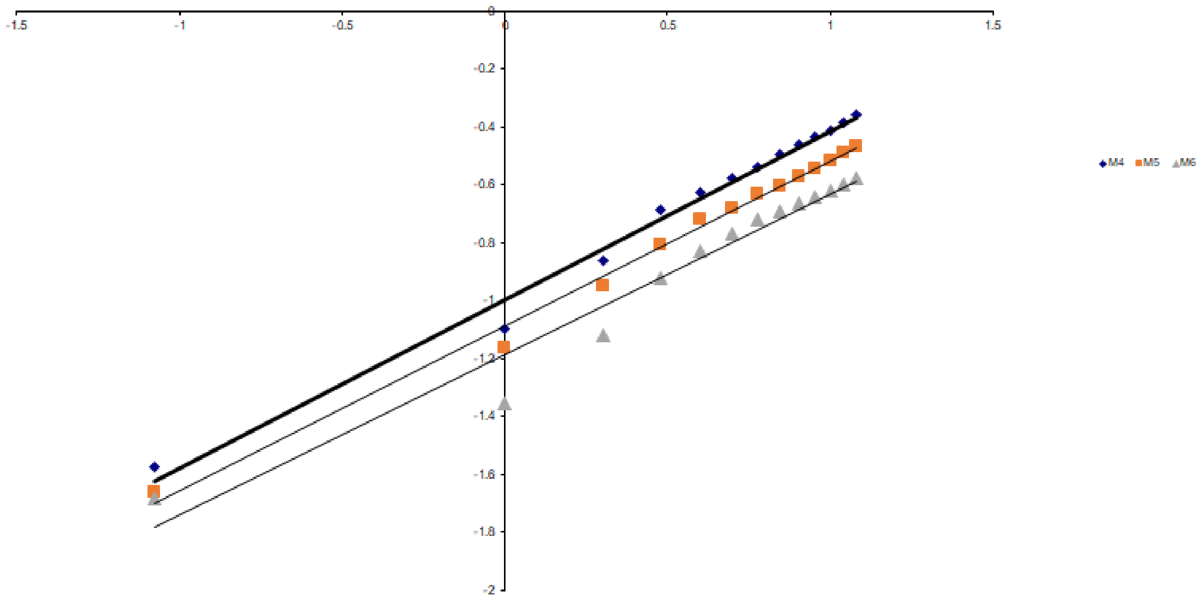

Figure S8. The release data after fitting to the Korsmeyer-Peppas model

----
